# Platelet-Rich Plasma (PRP) and Injectable Platelet-Rich Fibrin (i-PRF) in the Non-Surgical Treatment of Periodontitis—A Systematic Review

**DOI:** 10.3390/ijms25126319

**Published:** 2024-06-07

**Authors:** Wojciech Niemczyk, Katarzyna Janik, Jacek Żurek, Dariusz Skaba, Rafał Wiench

**Affiliations:** 1 Department of Periodontal Diseases and Oral Mucosa Diseases, Faculty of Medical Sciences in Zabrze, Medical University of Silesia, Pl. Traugutta 2, 41-800 Zabrze, Poland; dskaba@sum.edu.pl (D.S.); rwiench@sum.edu.pl (R.W.); 2 Specialist Medical Practice, Polne Wzgórze 11 Street, 32-300 Olkusz, Poland; kontakt@jacekzurek.pl

**Keywords:** root planing, platelet-rich plasma, platelet-rich fibrin, periodontics, periodontitis, injections, dental scaling, periodontal debridement, blood platelets

## Abstract

The gold standard in the non-surgical treatment of periodontitis is scaling and root planing (SRP). In recent years, the use of autogenous platelet concentrates has spread over many specialties in dentistry and, thus, has also been gaining popularity in periodontal treatment. Its two main fractions are platelet-rich plasma (PRP) and platelet-rich fibrin (PRF), which, since 2014, can also be used via injection as injectable platelet-rich fibrin (i-PRF). The authors conducted a comprehensive systematic review in accordance with the PRISMA 2020 guidelines. It involved searching PubMed, Embase, Scopus, and Google Scholar databases using the phrases (“Root Planing” OR “Subgingival Curettage” OR “Periodontal Debridement”) AND (“Platelet-Rich Plasma”). Based on the authors’ inclusion and exclusion criteria, 12 results were included in the review, out of 1170 total results. The objective of this review was to ascertain the impact of utilizing PRP and i-PRF in SRP. The results revealed that both the incorporation of PRP and i-PRF were found to be significantly associated with are duction in gingival pocket depth and again in clinical attachment level; however, i-PRF demonstrated superiority in improving clinical parameters. Furthermore, i-PRF demonstrated notable bactericidal efficacy against *Porphyromonas gingivalis*. On the other hand, PRP proved inferior to an Nd:YAG laser in clinical parameter improvement; however, it demonstrated significant efficiency as well. This literature review led the authors to the conclusion that autologous platelet concentrates might be competent agents for improving the therapeutic outcomes of SRP.

## 1. Introduction

Periodontal disease is a collective term used to describe a number of pathological conditions characterized by the degeneration and inflammation of the gums, periodontal ligaments, alveolar bone, and dental cementum [1]. This process is thought to be initiated by the interaction between dysbiotic microbial communities and aberrant immune responses within the gingival and periodontal tissues themselves [2,3,4,5]. The most recent classification divides periodontitis into four stages numbered from 1 to 4 and graded from A to C [6]. The aetiology of periodontal disease is primarily attributable to oral bacterial infections [7,8,9]. Non-surgical periodontal treatment (NSPT) and surgical periodontal treatment (SPT) are conventional procedures with regard to the control of infection, the reduction in probing pocket depth (PPD), and the clinical attachment level (CAL) gained. Currently, a minimally invasive approach involving proactive procedures is preferred. Probiotics as well as paraprobiotics used in adjunctive therapy are effective for reducing the bacterial load [10,11,12]. The maintenance of periodontal health is dependent upon the control of plaque [13,14,15]. It is widely accepted that scaling and root planing (SRP), a non-surgical treatment for periodontitis, is the gold standard for this indication. Its efficacy has been well documented in several systematic reviews [16,17,18,19]. Although numerous approaches to bone tissue engineering have historically concentrated on synthetic materials (such as polymers or hydrogels), contemporary methodologies are increasingly incorporating natural materials due to their inherent biological properties, exemplified by autologous bone grafting [20]. In the last few years, the use of blood concentrates has become increasingly prevalent in dentistry. These autologous treatments have been shown to facilitate natural healing, accelerate tissue regeneration, and provide patients with a more comfortable postoperative outcome [21,22,23,24]. Platelet-rich plasma (PRP) is an autologous blood product derived from the plasma fraction, which is created through the centrifugation process of whole blood. It is defined as having a platelet concentration above that of normal physiological levels [25,26]. The platelets present in PRP carry granules containing a significant number of active biomolecules. Upon activation, these biomolecules are released and subsequently stimulate the natural healing cascade [27,28]. Platelets are essential for the process of wound healing. Once activated, they release a range of growth factors, including platelet-derived growth factor (PDGF), transforming growth factor (TGF-β), and insulin-like growth factor I (IGF-I). Additionally, platelets secrete fibrin, fibronectin, and vitronectin, which act as a matrix for connective tissue and as adhesion molecules for more efficient cell migration. Consequently, they play a pivotal role in cell proliferation, collagen synthesis, and osteoid formation [29]. The use of PRP therapies has been documented for over three decades, during which time there has been considerable research interest in their potential for use in regenerative medicine [26,30,31]. The utilization of platelet-rich fibrin (PRF) has demonstrated a multitude of advantages over platelet-rich plasma (PRP) across numerous disciplines in the field of medicine. However, prior to 2014, PRF remained clinically available solely in its solid clotted form. The implementation of modifications to centrifugation protocols and tube technology has led to the advancement of PRF being available in a liquid injectable form (i-PRF) [32,33,34]. A key benefit of i-PRF is its ability to consistently release a range of growth factors, such as PDGF, TGF-β and IGF-I [35,36,37], which in turn promotes cell migration by inducing the expression of key proteins such as type I collagen and transforming growth factor mRNA [33,38]. The authors of this study noted that a systematic review examining the efficacy of PRP and i-PRF in the context of SRP is lacking. The extant literature on this subject displays a lack of consistency in the results produced. This has motivated the authors to undertake a comprehensive review in order to determine which findings are the most reliable. The objective of this study was to ascertain whether the utilization of PRP in SRP enhances the efficacy of short- and long-term treatment outcomes.

## 2. Materials and Methods

### 2.1. Focused Question

A systematic review was conducted in accordance with the PICO framework, as follows: in patients with chronic periodontitis (Population), does the adjunctive use of autologous platelet concentrates in conjunction with SRP (Intervention) result in a more efficacious improvement of clinical parameters (Outcome) compared to SRP alone (Comparison)?

### 2.2. Search Strategy

The review was conducted in accordance with the Preferred Reporting Items for Systematic Reviews and Meta-Analyses (PRISMA 2020) guidelines [39]. The electronic literature search conducted included the MEDLINE (PubMed) database, Embase, Google Scholar, and Scopus from inception until 16 April 2024. The keywords used in the searches of Scopus, Embase, and Google Scholar databases were (“Root Planing” OR “Subgingival Curettage” OR “Periodontal Debridement”) AND (“Platelet-Rich Plasma”). The terms used in the PubMed database search were ((((“Root Planing”[Mesh]) OR “Subgingival Curettage”[Mesh]) OR “Periodontal Debridement”[Mesh]) AND “Platelet-Rich Plasma”[Mesh]). In addition, the authors conducted a “snowball” search to identify further studies. This involved searching the reference lists of publications that had been deemed eligible for full-text review. Furthermore, Google Scholar was used to identify and verify citing studies. An additional inclusion criterion limited the electronic search to the English language. In order to minimize the risk of bias when searching for an article, the authors elected not to implement an electronic limitation in the form of randomized control trials. This was due to the fact that the tagging of papers is not always accurate, and the most recent papers may not yet have been tagged. The databases were searched by three authors, each of whom searched separately using the same search terms. After searching and selecting potential studies for inclusion in the review, all the authors jointly assessed whether the study in question met all the inclusion criteria. In order to collate the data from the included studies, the two authors conducted a joint search of the literature, with the objective of identifying the desired data.

### 2.3. Selection of Studies

The objective of this systematic review was to assess the efficacy of alternative approaches to periodontal treatment, specifically the use of autologous platelet concentrates in lieu of standard PRF. The hypothesis was that autologous platelet concentrates (APCs) would facilitate greater pocket depth reduction in the context of non-surgical periodontal therapy. The criteria for the inclusion of articles in and exclusion of articles from this review are presented in Table 1.

### 2.4. Risk of Bias in Individual Studies

In the initial phase of the study selection process, each reviewer individually assessed titles and abstracts in order to mitigate potential biases in the evaluation process. Cohen’s к test was employed as a tool to quantify the level of inter-reviewer agreement [40]. Any discrepancies regarding the inclusion or exclusion of a study in the review were discussed by the authors until a consensus was reached.

### 2.5. Quality Assessment

Two reviewers (W.N. and K.J.) conducted independent screenings of the included studies to assess their quality. The evaluation of study design, implementation, and analysis included the following criteria: double blinding of the study, random allocation of study participants and in the case of split-mouth studies, random selection of quadrants, clearly defined inclusion and exclusion criteria, whether the study had a split-mouth design, adequately balanced control/study groups within 10% of the participants, the calculated and necessary number of patients/pockets required for the study, accurately determined severity of periodontitis among the study patients, and a precise method of obtaining and administering the APCs used in the study. A score of 0–3 points indicated a high risk, 4–6 points denoted a moderate risk, and 7–9 points indicated a low risk. Discrepancies in scoring were resolved via discussion until a consensus was reached.

### 2.6. Risk of Bias across Studies

The scores for each study were calculated, and an overall estimated risk of bias (low, moderate, high) was determined for each included study, in accordance with the recommendations set forth in the Cochrane Handbook for Systematic Reviews of Interventions [41].

Of the 12 articles, 10 were identified as having a low risk of bias, and two were identified as having a moderate risk. The study with the highest risk of bias scored 5 out of 9 points, while one study scored the maximum number of points. Only one study lost 7 points, one study gained 6 points, and two gained 7 points each. No studies were excluded on the basis of low quality (high risk of bias), as the missing information was deemed non-essential for the thoroughness of the review. A single point was awarded in the event of a positive response. Conversely, no further points were allocated in the case of a negative or uncertain response. The assessment of the risk of bias was categorized as low, moderate, or high. The precise risk of bias for each included study is outlined in Table 2.

### 2.7. Data Extraction

The following information was extracted from each study: the mean age of the patients, standard deviation, age range, gender distribution, type of APCs used in the study, site of injection, indicators for evaluating outcomes, results, and the period of follow-up. In addition, the country in which the study was conducted was verified, as well as whether the study was conducted in a university centre and whether the study had a split-mouth design.

## 3. Results

### 3.1. Study Selection

**Figure 1:**
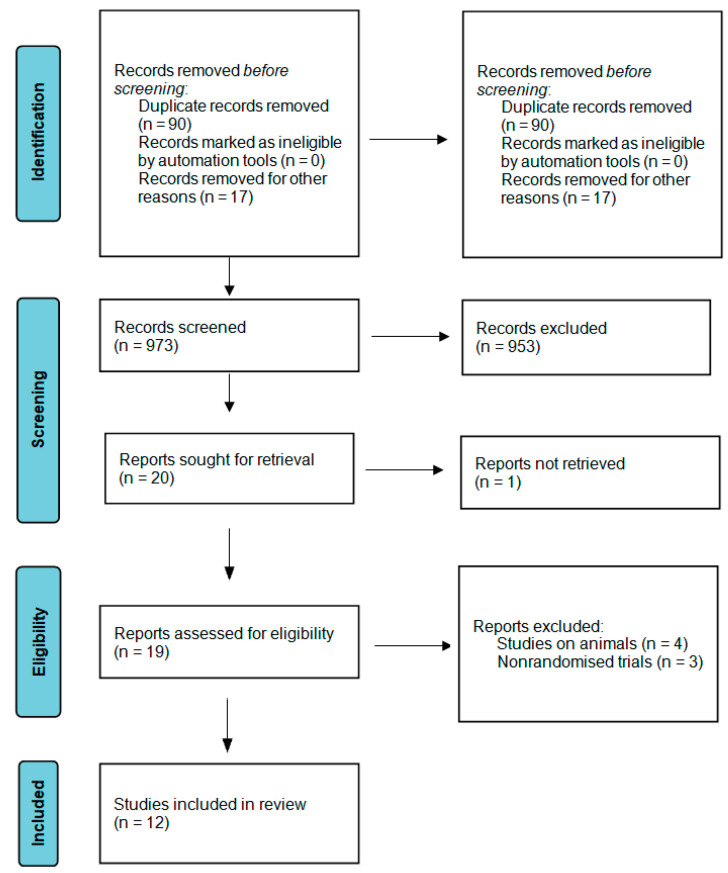
Prisma 2020 flow diagram.

A flowchart representing the research approach in accordance with the PRISMA statement [39] is displayed in Figure 1. A primary search of the databases yielded 1170 results. After the removal of duplicated studies, 973 studies were selected for the title and abstract screening, and after the screening, 953 studies were excluded as they did not fulfil the inclusion criteria [Table 1]. The remaining 20 studies were selected for full-text screening, and out of the 19 studies that the researchers had access to, 12 articles were selected, as they met the eligibility criteria and were included in the review, including 2 studies using PRP and 8 studies using i-PRF, with 1 study using both of them and 1 study using plasma rich in growth factors (PRGF).

All of the 12 included studies were randomized controlled trials that were published between 2014 and 2024. Only one of them was published before 2020. The number of study participants ranged from 6 to 87. Of the 12 studies, 9 had a split-mouth design and 3 were double-blinded. Further details can be found in Table 3.

### 3.2. General Characteristic of the Included Studies

In total, 8 out of every 12 studies were conducted after the requisite number of patients had been calculated in advance. A total of 10 of the 12 studies accurately determined the severity of periodontitis, with 2 employing the previously established classification system [54] and 10 utilizing the most recently introduced system [55]. Notably, 2 studies did not provide the distribution of subjects by sex, while an additional 2 studies lacked information about the mean age with its associated standard deviation. However, all but 3 studies offered the precise age range encompassing the patients under study. The collected data are categorised in Table 4.

Out of the twelve studies, two investigated the use of PRP, one study used PRGF, eight involved i-PRF, including one case of red i-PRF, and the remainder were not specified. There was also study involving both PRP and i-PRF. Each of the studies considered different clinical parameters for assessment, but all of them investigated probing pocket depth (PPD), and only one did not consider clinical attachment level (CAL). Follow-up periods ranged from six weeks to six months. The majority of authors employed APCs interproximally, from the bottom up to the overflow of the coronal part. However, only Vučković et al., utilized individually formed occlusal splints for this purpose. A summary of the key points from each article is presented in Table 5.

### 3.3. Main Study Outcomes

CAL and PPD were investigated in all i-PRF studies [42,43,44,45,46,47,48,49,50,51,52,53]. Each showed reductions in pocket depth and improvements in CAL rates. The study by Torumtay Cin et al. [52] additionally demonstrated that gingival recession values were significantly lower than in the control group, and, additionally, VEGF and IL-10 levels were significantly higher than in the control group 14 days after treatment. Additionally, TNF-α levels were significantly lower in the test group on days 7 and 14. Mazloum et al. [48] compared not only i-PRF to SRP alone but also i-PRF to HA. Both the i-PRF and HA groups demonstrated a significant reduction in PD and gain in CAL. By contrast, no significant differences in these parameters were observed between the HA and i-PRF groups. By contrast, Khallaf et al. [47] compared the use of piroxicam gel and i-PRF 2 weeks after the SRP procedure. While both groups demonstrated improvement in the evaluated parameters, the results were significantly more favourable in patients who had i-PRF administered. Shunmuga et al. [51] conducted a study on patients diagnosed with type 2 diabetes, and concluded that i-PRF application toSRP in these patients provided similar benefits to saline application to SRP. Elarif et al. [45] demonstrated that patients treated with SRP in conjunction with i-PRF exhibited greater gingival pocket reduction in comparison to the control group treated with SRP alone and to the group treated with SRP in conjunction with antimicrobial photodynamic therapy (aPDT). Furthermore, the same group treated with i-PRF exhibited a superior bactericidal effect against *Porphyromonas gingivalis* in comparison to the other groups. Conversely, the group treated with aPDT demonstrated a greater gain in clinical attachment level (CAL) and a reduction in gingival index (GI) compared to the group treated with i-PRF. Agarwal and Dev Gupta [42] conducted a double-blinded trial comparing PRP to placebo after SRP. The results demonstrated the long-term benefits of PRP with non-surgical periodontal pocket treatment. The mean CAL gain was 2.40 ± 0.4 mm for the control sites and 2.68 ± 0.5 mm for the experimental sites. A study conducted by El Sharaki et al. [46] compared the performance of the Nd:YAG laser to PRP after SRP. The results demonstrated that clinical parameters such as PD, GI, CAL, PI, and radiographic bone defect significantly improved with the Nd:YAG laser, which was found to be superior to PRP treatment. Nevertheless, the use of PRP also showed a significant improvement in clinical parameters. The study by Amin et al. [44] was the only study to compare the effects of PRP and i-PRF. In their study, 30 patients underwent SRP in conjunction with PRP, while 30 patients underwent SRP in conjunction with i-PRF. Both patients in the PRP and i-PRF groups demonstrated superior outcomes compared to the control group undergoing SRP alone. A statistically significant difference was observed in mean clinical attachment loss between the three groups, with the i-PRF group demonstrating the highest reduction from baseline (84.80 ± 17.10), followed by the PRP group (82.84 ± 11.63) and the control group (74.07 ± 6.84). A statistically significant difference in pocket depth was observed between baseline and 1, 2, and 3 months in both the PRP and i-PRF groups (*p* < 0.001). Consequently, a statistically significant decrease in PD from baseline was evident in all test and control sites. The percentage reduction in PD from baseline to 1, 2, and 3 months demonstrated a statistically significant difference in mean probing pocket depth across the three groups (*p* < 0.001). The i-PRF group exhibited the highest reduction, followed by the PRP group, and then the control group. Panda et al. [49] were the sole researchers to utilize PRGF in their study. They demonstrated that the incorporation of PRGF technology in non-surgical periodontal therapy, through a single application to periodontal pockets as an adjunct to SRP in patients with chronic periodontitis, was efficacious in reducing pocket depth and increasing clinical attachment levels.

## 4. Discussion

The results of the studies included in the review are almost unequivocal in their support of the use of APCs during SRP. The only two studies that did not show a positive correlation was that of Albonni et al., and Shunmug et al., which found no additional benefit from the use of i-PRF compared to the saline solution. It is also important to note that the study by Shunmug et al., is the only study in which the inclusion criterion was patients’ systemic disease in the form of type 2 diabetes, which might have introduced bias into the results, compared to healthy patients. Furthermore, an additional study demonstrated inferior outcomes when PRP was employed in comparison to another study group that had undergone Nd:YAG laser therapy. The study by Sharaki et al., concluded that although PRP significantly enhanced clinical parameters following SRP, the laser performed significantly better. By contrast, the opposite results were obtained by Acatrinei et al. [56] in their non-randomised study, where it was patients receiving PRP who showed greater pocket shallowing than patients with SRP accompanied by laser therapy. As an exemplary study, the authors adopted the Agarwal and DevGupta study, as it was the only one to score the maximum number of points when determining the risk of bias, and it was conducted on the largest number of patients (87). The lack of homogeneous methodology in the available studies precludes the ability to draw definitive conclusions and assess if i-PRF and PRP give better clinical outcomes. It can be concluded from the Amin et al., study that i-PRF gives better results than PRP, as it was the only study to compare these APCs. However, it should be noted that this study was conducted on a relatively large number of patients (70) but was not double-blinded. Such outcomes may also reinforce other studies that compare the effects of i-PRF to PRP. It has been demonstrated that the utilisation of i-PRF, employing the low-speed centrifugation concept, significantly enhances chondrocyte activity and further optimises cartilage regeneration in comparison to PRP. The histological findings revealed accelerated and superior cartilage regeneration within four weeks postoperatively when i-PRF was employed, with the results maintained at 12 weeks [57]. Similar outcomes were observed when examining the impact of these APCs on osteoblast behaviour. The findings indicated that the naturally formulated i-PRF exhibited a more favourable effect than traditional PRP with anti-coagulants [58]. Furthermore, Miron et al., demonstrated that i-PRF was capable of releasing higher concentrations of various growth factors and inducing higher fibroblast migration and expression of PDGF, TGF-β, and collagen 1 [59]. Additionally, i-PRF possesses several advantages over PRP. Primarily, i-PRF is produced via a single centrifugation protocol, which represents a significant advantage over PRP. Secondly, i-PRF contains a higher concentration of leukocytes, which is beneficial in certain applications [57]. A study by Elarif et al., demonstrated the significant effect of i-PRF in reducing the titre of *Porphyromonas gingivalis* bacteria in comparison to the control group. A study comparing the antimicrobial effect of APCs in the form of PRF, i-PRF, and PRP revealed that in the case of *Porphyromonas gingivalis*, i-PRF exhibited the widest zone of inhibition, which was significantly wider than that of PRF. Moreover, PRP exhibited a significantly wider zone of inhibition in comparison to PRF. In the case of *Aggregatibacter actinomycetemcomitans*, PRP demonstrated a wider zone of inhibition, which was significantly wider than that of PRF and i-PRF [60]. Similar outcomes were observed by Pham in his investigation comparing the antimicrobial efficacy of advanced PRF plus (A-PRF+) to i-PRF against *P. gingivalis*. The findings demonstrated that both A-PRF+ and i-PRF exhibited antibacterial properties against *P. gingivalis*, with i-PRF exhibiting a more pronounced effect [61]. A study by Karde et al., comparing the inhibitory effects of PRP, PRF, and i-PRF demonstrated that i-PRF exhibited the greatest zone of inhibition around oral microbiota, with an average of 1.42 ± 0.25 cm. The order of zone of inhibition from highest to lowest was i-PRF > PRF > PRP. In a single study, the antimicrobial efficacy of PRP and PRF was evaluated against *Porphyromonas gingivalis* and *Aggregatibacter actinomycetemcomitans* [29]. Yeaman put forth a hypothesis suggesting that direct interactions between platelets and microorganisms, participation in antibody-dependent cell cytotoxicity, and engulfment by entrapped white blood cells within PRF could result in direct bacterial killing. In addition to this, it has been posited that the release of myeloperoxidase and the activation of antioxidant responsive elements and antigen-specific immune responses could occur [62]. In addition, various mechanisms have been proposed to explain the antibacterial effect of platelet-derived preparations. These include the generation of oxygen metabolites, including superoxide, hydrogen peroxide, and hydroxyl free radicals; the binding, aggregation, and internalisation of microorganisms, thereby enhancing the clearance of pathogens from the bloodstream; and the release of an array of potent antimicrobial peptides [63].

There are several studies, reaching similar results, that confirm the findings of this article [29,59,64,65,66,67,68,69,70,71,72,73]. Özcan et al. [64] observed that using PRF in the procedures of SRP provided significantly greater pocket reduction, higher clinical attachment gain, and less gingival recession than the control group at 3 and 6 months. Moreover, it led to higher levels of transforming growth factor-β and collagen-1 in gingival crevicular fluid throughout the first two-week period, which was indicative of enhanced initial healing process. In another trial, a reduction in the mean values of PI, GI, BOP, PPD, and RAL 1 month post-application of the PRP was noted, as well as a marked reduction in the lymphocyte count at baseline and after 1 month, with a highly significant difference between the measurements [65]. Similar results were obtained by Narendran et al. [66] in terms of reduction in PD and clinical attachment gain compared to the control sites at the 2- and 3-month follow-up. The findings of the study conducted by Aydinyurt et al. [67] on rats indicated that the i-PRF application was as efficacious as SRP in reducing bone loss, modulating the inflammatory process, and regulating cytokines in the context of experimental periodontitis. Furthermore, a comparison of PRP, i-PRF, and PRF in terms of the growth factors they contain and their regenerative potential is pertinent to this review. Such a comparison was carried out by Karde et al., who demonstrated that i-PRF and PRP exhibited a 503% and 464% increase in platelet numbers, respectively. In comparison, the PRF clot exhibited a platelet concentration of approximately 87% when compared to whole blood. It is well established that preparations with higher platelet counts release more growth factors [29]. Miron et al., demonstrated that, in general, PRP had a higher early release of growth factors, whereas i-PRF showed significantly higher levels of total long-term release of these factors [59,68]. Furthermore, the study by Iozon et al., demonstrated that five percent i-PRF stimulated gingival mesenchymal stem cell proliferation after seven days of culture but not after three days. This suggests that a certain time is needed for growth factors to induce local stimulation. Therefore, the use of slow-releasing growth factor products, such as i-PRF, is crucial to ensure biological stimulation for clinical purposes. Additionally, an excessively high concentration of i-PRF could impair osteogenesis [69]. It should also be noted that PRP, despite being an autologous preparation, necessitates the addition of thrombin and calcium for its activation, with the potential for the development of immunogenic responses against the clotting factors V, XI, and thrombin, which could have an adverse impact on the coagulation process, as well as trigger an immune reaction. PRF, the second-generation platelet concentrate introduced by Choukroun (2001) [70], is a straightforward preparation process that offers favourable handling characteristics. It does not involve the use of bovine thrombin or an anticoagulant, which considerably reduces the biochemical handling of blood and its associated risks. PRF itself contains physiologically available thrombin, which is responsible for the slow polymerisation of fibrinogen into fibrin, resulting in a physiologic architecture favourable to wound healing. This fibrin network protects the growth factors from proteolysis. Furthermore, PRF facilitates the development of microvascularisation, thereby enhancing the efficiency of cell migration [71]. The introduction of i-PRF was based on a similar concept to that of PRF, with a notable advantage in the injectable form of the latter, which can be used independently or in combination with a variety of biomaterials. Its procedure is founded on the principle that slower and shorter centrifugation spins result in a greater presence of regenerative cells with higher concentrations of growth factors [72]. In the study conducted by Karde et al., it was observed that the i-PRF method yielded the highest platelet count, which was statistically significant [29]. This could be attributed to the low centrifugation speed and time, which resulted in a higher number of platelets. Ghanaati et al., introduced the “low-speed concept” for blood centrifugation, whereby lower centrifugation speeds were shown to contain higher numbers of cells, including leukocytes, before the formation of a fibrin clot [72]. A notable aspect of the utilisation of APCs in patients diagnosed with periodontitis is the absence of any statistically significant disparity observed between the growth factors VEGF, IGF-1, TGF-β1, platelet-derived growth factor subunit B (PDGF-BB), and the epidermal growth factor derived from healthy patients and those with periodontitis [73]. Future research should also focus on comparing platelet factors with other forms of treatment, such as hyperbaric oxygen therapy [74] or ozonised gels [75], in order to identify the most effective form of platelet factors and the most appropriate form of treatment for patients.

The most significant limitation preventing the drawing of clear conclusions is the quantity of work that has been produced thus far. The existing works are characterized by small study groups, and the methods used to evaluate the results are not heterogeneous, as different authors have chosen different indicators to evaluate. An important burden on the results of the work is the use of probes calibrated every 1 mm, which translates into inaccurate measurements. A further limitation of the presented studies is the relatively short follow-up period, which ranged from six weeks to six months. Nevertheless, the authors only included papers with the lowest risk of bias in the analysis, which is a strength of this article. Nine of the twelve studies were conducted with a split-mouth design, where the same patients constituted the same control and study group. The search for articles was conducted independently by three authors, with broad search criteria employed to minimize the risk of omitting important articles for the review. The inclusion and exclusion criteria were applied rigorously, resulting in a limited number of articles included in the review. However, the literature review was only in English, which may have limited the number of studies found. The results of the review, although not yet definitive due to the limited number of patients studied, are certainly promising. In almost all studies, the parameters studied showed improvement compared to the control groups. The use of PRP, i-PRF, and PRGF is much more convenient than the well-known PRF, due to the fact that PRP and i-PRF are used by injection and PRGF is in gel form. It is recommended that randomized studies be conducted to compare the four APCs in terms of clinical outcomes and ease of use. Furthermore, authors of future papers should endeavour to obtain data from larger study samples and with longer follow-up periods. Also, an important aspect that was not developed in each of the articles was the mode of administration of APCs. It is therefore recommended that authors of future studies describe exactly where they administer the preparation and to which level of the pocket. In the studies cited, no differences were apparent between the modes of administration of PRP and i-PRF. It is essential that the data obtained take into account not only the number of patients studied, but also the number of pockets that were subjected to the study. This will ensure greater reliability of the results obtained, which can be more easily compared to others and create a relevant meta-analysis.

## 5. Conclusions

PRP, as well as i-PRF, show anti-inflammatory properties and promote healing process of the tissues of periodontium, due to their ability to release growth factors, as well as components of the extracellular matrix of connective tissue. Regardless of the limited amount of research, the findings of this study demonstrate that they are a helpful tool in the nonsurgical debridement of periodontal pockets during the treatment of chronic periodontitis. They present promising opportunities in treatment outcome variables in SRP procedures; however, further studies are crucial to facilitate the implementation of such agents on a wide scale.

## Figures and Tables

**Table 1:** Selection criteria for papers included in the systematic review.

Inclusion Criteria	Exclusion Criteria
Randomized controlled trials English language Fulltext available Human studies APCs in non-solid form Patients aged ≥18 years	Non-randomized trials Case reports/Case series Reviews Systematic reviews Meta-analysis Conference papers Letters to Editor Abstracts Animal studies Studies on smoking patients High risk of study bias Solid form of PRF Non-English language publications

**Table 2:** The results of the quality assessment and risk of bias across the studies.

	Study											
Criteria	Agarwal and Dev Gupta (2014) [42]	Albonni et al. (2021) [43]	Amin et al. (2022) [44]	Elarif et al. (2022) [45]	El Sharaki (2023) [46]	Khallaf et al. (2024) [47]	Mazloum et al. (2023) [48]	Panda et al. (2020) [49]	Rakhewar et al. (2021) [50]	Shunmuga et al. (2023) [51]	Torumtay Cin et al. (2023) [52]	Vučković et al. (2020) [53]
Random allocation	1	1	1	1	1	1	1	1	1	1	1	1
Inclusion/exclusion criteria clearly defined	1	1	1	1	1	1	1	1	1	1	1	1
Split-mouth study type	1	1	1	0	1	0	0	1	1	1	1	1
Balanced study groups	1	1	0	1	1	1	1	1	1	1	1	1
Double-blinded study	1	1	1	0	0	0	0	1	0	0	0	0
Calculated study group	1	0	1	1	0	1	1	1	0	1	1	0
Precisely defined severity of periodontitis	1	1	1	1	0	1	1	1	1	1	1	0
Clear method of obtaining APCs	1	1	1	1	1	1	1	1	1	1	1	1
Well-defined method of administration of APCs	1	1	1	1	0	1	1	0	0	1	1	1
Total	9	8	8	7	5	8	7	8	6	8	8	8
Risk of bias	Low	Low	Low	Low	Moderate	Low	Low	Low	Moderate	Low	Low	Low

**Table 3:** A general overview of the studies.

Author and Year	Country	Setting	Study Design	Split-Mouth
Agarwal and Dev Gupta (2014) [42]	India	University	Double-blinded Randomized Controlled Trial	Yes
Albonni et al. (2021) [43]	Syria	University	Double-blinded Randomized Controlled Trial	Yes
Amin et al. (2022) [44]	Egypt	University	Randomized Controlled Clinical Trial	Yes
Elarif et al. (2022) [45]	Egypt	University	Randomized Controlled Clinical Trial	No
El Sharaki (2023) [46]	Egypt	University	Randomized Controlled Clinical Trial	Yes
Khallaf et al. (2024) [47]	Egypt	University	Randomized Controlled Clinical Trial	No
Mazloum et al. (2023) [48]	Lebanon	University	Randomized Controlled Clinical Trial	No
Panda et al. (2020) [49]	India	University	Double-blinded Randomized Controlled Trial	Yes
Rakhewar et al. (2021) [50]	India	University	Randomized Controlled Clinical Trial	Yes
Shunmuga et al. (2023) [51]	India	University	Randomized Controlled Clinical Trial	Yes
Torumtay Cin et al. (2023) [52]	Turkey	University	Randomized Controlled Clinical Trial	Yes
Vučković et al. (2020) [53]	Serbia	University	Randomized Controlled Clinical Trial	Yes

**Table 4:** Characteristics of patients by study.

Sample Characteristics							
Author/Year	Sample Size Calculation	Study Population	Patients	Sex		Age (Years)	
				Female	Male	Mean (±SD)	Range
Agarwal and Dev Gupta (2014) [42]	Yes	Moderate to severe chronic periodontitis	87	39	48	45 ± 4.6	30–50
Albonni et al. (2021) [43]	No	Periodontitis stage II to III with grade B to C	15	3	12	45	37–64
Amin et al. (2022) [44]	Yes	Periodontitis stage II to III with grade B to C	70	18	22	34.0 ± 7.95	No data
Elarif et al. (2022) [45]	Yes	Stage III grade B periodontitis	39	26	13	40.4 ± 4.38	30–55
El Sharaki (2023) [46]	No	Chronic periodontitis	30	No data			25+
Khallaf et al. (2024) [47]	Yes	Stage III grade B periodontitis	6	6	0	No data	30–60
Mazloum et al. (2023) [48]	Yes	Stage III periodontitis grade A or to B	63	33	30	51.8 ± 10.8	20–60
Panda et al. (2020) [49]	Yes	Periodontitis, stage III, grade A or B	26 (22 evaluated after 6 months)	11	15	35.8 ± 12.7	30–50
Rakhewar et al. (2021) [50]	No	Moderate to severe chronic periodontitis	10	No data		39.2 ± 4.1	35–48
Shunmuga et al. (2023) [51]	Yes	Stage III, grade C periodontitis Patients with type 2 diabetes	23	13	10	51.1 ± 11.72	30–75
Torumtay Cin et al. (2023) [52]	Yes	Periodontitis stage 3,grade B	17	7	10	37.4 ± 5.84	No data
Vučković et al. (2020) [53]	No	Patients with chronic periodontitis	24	14	10	37.29 ± 10.2	22–64

**Table 5:** Detailed characteristics of the studies included in this review.

Author/Year	Treatment	Crucial Inclusion Criteria	Injection Site and Method of Administration	Evaluation	Results	Follow-Up Period
Agarwal and Dev Gupta (2014) [42]	PRP	Pockets ≥5 mm associated with single-rooted teeth, approximately similar radiographic angular bone defects ≥3 mm	Bottom of the pocket until the pocket was overfilled	PPD CAL PI mSBI	- Statistically significant changes in parameters in both groups from baseline to 6 months - Significantly greater clinical attachment gain (*p* > 0.05) in the test group - Mean CAL gain for control sites 2.40 ± 0.4 mm and for test sites 2.68 ± 0.5 mm	6 months
Albonni et al. (2021) [43]	i-PRF	Bilateral periodontal pockets (≥5 mm)	Bottom of the pocket until the pocket was overfilled	BOP PI PPD CAL	- Statistically significant decreases in PI (*p* = 0.001), BOP (*p* = 0.001 for both groups), PPD (*p* = 0.001 and *p* = 0.000 for test and control groups, respectively), and CAL (*p* = 0.015 and *p* = 0.001 for test and control groups, respectively) in both test and control groups - No statistically significant differences for inter-group comparisons in any of the clinical indices (*p* > 0.05).	3 months
Amin et al. (2022) [44]	PRP i-PRF	Bilateral interproximal defect, PPD ≥ 5 mm on a minimum of 2 teeth, CAL 3 mm or more than 5 mm	Gingival sulcus until the blanching and fullness of gingiva was noted	PI BI GI PPD CAL	- Statistically significant decreases in mean PPD, CAL - Higher reduction in the iPRF group followed by the PRP group then the control group (*p* < 0.001)	3 months
Elarif et al. (2022) [45]	i-PRF	CAL more than 4 mm, PPD more than 5 mm, Bone loss extends to the middle or apical third of affected roots.	Deepest pocket intra-sulcularly	GI PI CAL PPD Bactericidal effect against PG	- i-PRF group demonstrated a notable decline in the proportion of Pg at the conclusion of one month in comparison to the other groups - Statistically significant higher reduction inGI and gain of CAL in the aPDT group in contrast to the i-PRF group showing higher reduction inPPD	3 months
El Sharaki (2023) [46]	PRP	Bilateral periodontal pockets (≥ 5 mm) and radiographic evidence of bone loss	Periodontal pockets	PPD GI CAL PI Radiographic bone defect	- Significantly larger decrease in all clinical parameters in the Nd:YAG laser group than the PRP group (at both the 1-month and 6-month post-treatment evaluations (*p* < 0.001) - Reductions in PPD, GI, CAL, PI, and radiographic bony defects in Nd:YAG laser group	6 months
Khallaf et al. (2024) [47]	i-PRF	Proximal tooth surface shoving PPD ≥ 6 mm	Bottom of the pocket until the pocket was overfilled	PPD CAL BOP Immunologically—levels of matrix metalloproteinases-8	- Significant improvement in all clinical and immunological parameters in both groups - Higher improvement in all assessed parameters in the i-PRF group than the piroxicam group at each follow-up time point	3 months
Mazloum et al. (2023) [48]	Red i-PRF	At least 4 periodontal sites with a PPD ≥ 6 mm. Radiographic evidence of bone loss and CAL ≥ 5 mm	Pocket at the point of interdental space	CAL PPD BOP GI PI	- Significant improvement in PI, GI, and BOP in all groups - The highest decrease inPPD in the HA group and the i-PRF group - A notable increase in CAL in the HA group and the i-PRF group, in contrast to control group showing no improvement	3 months
Panda et al. (2020) [49]	PRGF	PPD > 5 mm and presence of bleeding on probing	Deeper pockets	PPD RAL SBI	- Statistically significant higher reduction inPPD (*p* = 0.007) and gain of RAL (*p* = 0.021) in the PRGF group - Statistically significant difference for all parameters in the intra-group comparison - Significantly lower number of sites with PPD > 4 mm that required further treatment following the six-month follow-up period in PRGF group	6 months
Rakhewar et al. (2021) [50]	i-PRF	Minimum 2 sites with PPD ≥ 5 mm	Periodontal pocket	CAL PPD BOP PI	- The mean decrease inCAL from 6.2 ± 0.63 to 5.1 ± 0.65 in the test group, while in the control group from 6.3 ± 0.94 to 5.6 ± 0.69 - The total reduction inCAL in the test group 1.1 ± 0.31 and in the control group 0.7 ± 0.34 - Statistically significant higher reduction inBOP, PI and PPD in the test group	6 weeks
Shunmuga et al.(2023) [51]	i-PRF	≥5 mm PPD with attachment loss involving at least two interproximal sites	Bottom of the pocket until the pocket was overfilled	PI MGI PPD CAL GR	- The mean decrease inPPD and CAL from 6.30 ± 1.25 and 7.48 ± 1.75 at baseline to 3.48 ± 1.34 and 4.39 ± 1.67 at six months in control sites - The mean decrease inPPD and CAL from 6.57 ± 1.56 and 7.61 ± 1.69 to 3.39 ± 1.23 and 4.26 ± 1.81 at six months in test sites (*p* ≤ 0.0001) - No statistically significant differences between SRP + i-PRF and SRP + saline for clinical parameters improvement	6 months
Torumtay Cin et al. (2023) [52]	i-PRF	CAL ≥ 5 mm, PPD ≥ 6 mm, radiographic bone loss extending the mid-third of the root, and ≤4 teeth lost due to periodontitis	A small portion of i-PRF was injected into a selected inner epithelial layer of the periodontal pockets. Injections were applied subgingivally, starting at the bottom of the periodontal pocket and moving coronally, targeting the midpoint of the sulcus epithelium. The remaining i-PRF was injected into the gingival sulcus.	GI PI BOP PPD CAL GR Levels of: VEGF TNF-α IL-10 GCF	- Mean pocket reduction (PD) and clinical attachment (CAL) gain significantly higher in the test group than in the control group at follow-up visits (*p* < 0.05) - Gingival recession (GR) values significantly lower in the test group than in the control group - VEGF and IL-10 levels significantly higher in the test group than in controls at the 14th day, while TNF-α levels significantly lower in the test group at the 7th and 14th days	6 months
Vučković et al. (2020) [53]	i-PRF	(PPD) ≥ 5 mm on contralateral sides	The use of individually formed occlusal splints with periodontal pockets through perforations at the point of interdental space enabled the splint to be held in place for a longer period.	CAL GML PPD BOP PI	- The mean reduction inCAL from 1.97 ± 0.75 (0.25–3.31) to 1.07 ± 0.44 (0.12–1.78) - In the test group, the mean value decreased from 1.81 ± 0.66 (0.42–2.96) to 1.48 ± 0.55 (0.22–2.30), in contrast to the control group from 1.97 ± 0.75 (0.25–3.31) to 1.07 ± 0.44 (0.12–1.78) - Corresponding values for GML and PPD demonstrated a statistically significant difference between the groups (*p* = 0.040 and *p* = 0.006, respectively)	3 months

SRP—Scaling and Root Planing, PRP—Platelet-Rich Plasma, i-PRF—Injectable Platelet-Rich Fibrin, PPD—Probing Pocket Depth, CAL—Clinical Attachment Level, PI—Plaque Index, mSBI—Modified Sulcus Bleeding Index, GML—Gingival Margin Level, BOP—Bleeding on Probing, GI—Gingival Index, PG—*Porphyromonas gingivalis*, RAL—Relative Attachment Level, SBI—Sulcus Bleeding Index, GR—Gingival Recession, MGI—Modified Gingival Index, GCF—Gingival Crevicular Fluid, VEGF—Vascular Endothelial Growth Factor, TNF-α—Tumour Necrosis Factor-α, IL—Interleukin, PRGF—Plasma Rich in Growth Factors.
